# Evaluation of the outcomes of biliary-enteric reconstruction in robotic radical resection of hilar cholangiocarcinoma: a single-center propensity score matching analysis

**DOI:** 10.1038/s41598-024-65875-8

**Published:** 2024-06-27

**Authors:** Jie Liu, Changwei Dou, Jian Chen, Yi Lu, Lei Liang, Fangqiang Wei, Chengwu Zhang

**Affiliations:** General Surgery, Department of Hepatobiliary & Pancreatic Surgery and Minimally Invasive Surgery, Cancer Center, Zhejiang Provincial People’s Hospital (Affiliated People’s Hospital, Hangzhou Medical College), Hangzhou, 310014 Zhejiang China

**Keywords:** Robotic, Laparoscopic, Hilar cholangiocarcinoma, Biliary-enteric reconstruction, Propensity score matching, Cancer, Gastroenterology, Oncology

## Abstract

Although robotic radical resection for hilar cholangiocarcinoma (HCCA) has been reported in some large hepatobiliary centers, biliary-enteric reconstruction (BER) remains a critical step that hampers the operation’s success. This study aimed to evaluate the feasibility and quality of BER in robotic radical resection of HCCA and propose technical recommendations. A retrospective study was conducted on patients with HCCA who underwent minimally invasive radical resection at Zhejiang Provincial People’s Hospital between January 2016 and July 2023. A 1:2 propensity score matching (PSM), widely used to reduce selection bias, was performed to evaluate the outcomes, especially BER-related data, between the robotic and laparoscopic surgery. Forty-six patients with HCCA were enrolled; ten underwent robotic-assisted resection, while the others underwent laparoscopic surgery. After PSM at a ratio of 1:2, 10 and 20 patients were assigned to the robot-assisted and laparoscopic groups, respectively. The baseline characteristics of both groups were generally well-balanced. The average liver resection time was longer in the robotic group than in the laparoscopic group (139.5 ± 38.8 vs 108.1 ± 35.8 min, P = 0.036). However, the former had less intraoperative blood loss [200 (50–500) vs 310 (100–850) ml], despite no statistical difference (P = 0.109). The number of residual bile ducts was 2.6 ± 1.3 and 2.7 ± 1.2 (P = 0.795), and anastomoses were both 1.6 ± 0.7 in the two groups (P = 0.965). The time of BER was 38.4 ± 13.6 and 59.1 ± 25.5 min (P = 0.024), accounting for 9.9 ± 2.8% and 15.4 ± 4.8% of the total operation time (P = 0.001). Although postoperative bile leakage incidence in laparoscopic group (40%) was higher than that in robotic group (10%), there was no significant difference between the two groups (P = 0.204); 6.7 ± 4.4 and 12.1 ± 11.7 days were observed for tube drawing (P = 0.019); anastomosis stenosis and calculus rate was 10% and 30% (P = 0.372), 0% and 15% (P = 0.532), respectively. Neither group had hemorrhage- or bile leakage-related deaths. Robotic radical resection for HCCA may offer perioperative outcomes comparable to conventional laparoscopic procedures and tends to be advantageous in terms of anastomosis time and quality. We are optimistic about its wide application in the future with the improvement of surgical techniques and experience.

## Introduction

Hilar cholangiocarcinoma (HCCA) accounts for over 80% of all cholangiocarcinomas^1,2^ and generally has a poor survival rate following resection^3–5^. Radical resection is recognized as the only curative treatment for this disease. Although some retrospective studies have shown that laparoscopic radical resection of HCCA (LRRH) can achieve similar outcomes or even better than open surgery in lymph node dissection and intraoperative bleeding, which accelerate postoperative recovery for patients^6–8^, the minimal invasiveness of LRRH remains controversial^9–11^. In addition, the high technical requirements and long practice curves limit the application of HCCA resection using a laparoscopic approach. The inherent disadvantages of laparoscopy make biliary-enteric reconstruction (BER) a crucial rate-limiting step in LRRH. Compared to traditional laparoscopy, robotic surgical systems offer stable and highly flexible robotic arms and a high-definition magnified 3D lens, enabling precise surgical operations in narrow spaces^12,13^. However, there is currently no single clinical study on the advantages and disadvantages of robotic surgery for BER in the radical resection of HCCA. Therefore, we conducted a comparative study focusing on the rate and time percentage of BER completion and technical operability in laparoscopic and robotic approaches, as well as their influence on short-term postoperative complications.

## Material and method

### Patients’ cohort and data collection

**Figure 1:**
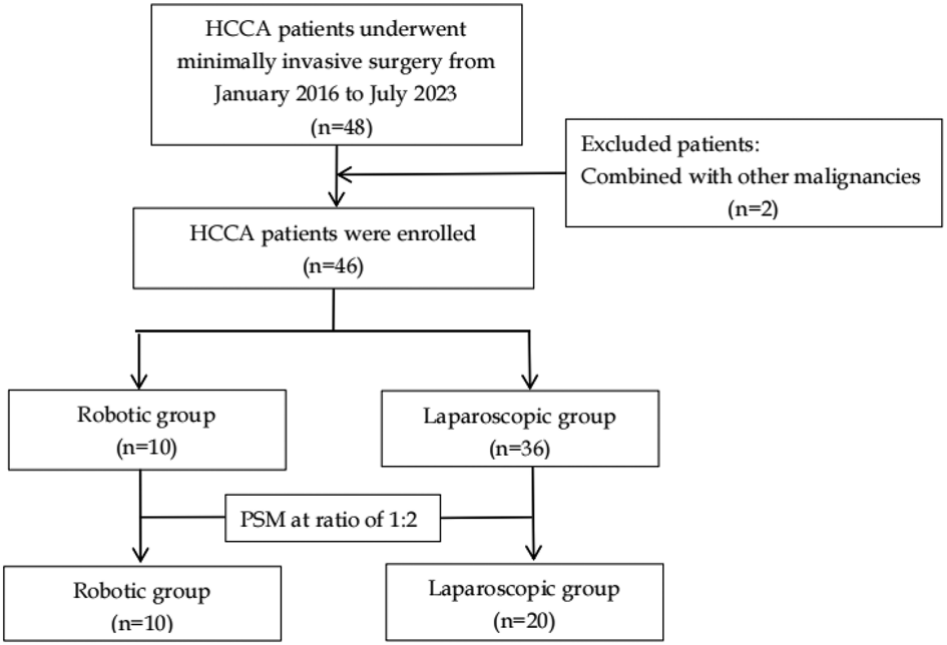
Flow chart of patients selection. *HCCA* hilar cholangiocarcinoma, *PSM* propensity-scored matching.

Data for a consecutive series of patients who underwent robotic or laparoscopic radical resection for HCCA at Zhejiang Provincial People’s Hospital between January 2016 and July 2023 were retrieved from a prospectively collected database. Patients enrolled in this study for minimally invasive resection met the following criteria: first, they must meet the indications for radical resection of the HCCA using a robotic or laparoscopic approach^6,14^. HCCA with metastases to other organs, intraperitoneal seeding, and exceeding regional lymph node metastasis were considered unresectable. Second, patients with vascular invasion were excluded, meaning no vascular resection or reconstruction was required. Finally, the patient tolerated the pneumoperitoneum and prolonged anesthesia. A flowchart is shown in Fig. 1.

Data were recorded using a standard case report form, and the Bismuth–Corlette classification of the patients was determined by a combination of preoperative imaging, intraoperative findings, and postoperative pathology. Outcomes including preoperative biochemical parameters, total operating time, liver resection time, intraoperative blood loss and transfusion, the total number of lymph nodes examined (TNLE), margin status (R0/R1/R2), drainage duration, postoperative hospital stay, and complications were assessed. The severity of postoperative complications was evaluated by Clavien–Dindo (CD) classification^15^, and CD ≥ III was defined as serious complications. BER-related data were recorded and evaluated in detail to satisfy the purpose of this study. The emphasis was on the amount of bile duct residue, the number of anastomoses, the method of suturing, and time consumption. The main complications were bile leakage, anastomotic stricture, anastomotic stones, and BER-related death.

### Preoperative management

Ultrasonography, computed tomography (CT), magnetic resonance cholangiopancreatography (MRCP), and 3-D image reconstruction were routinely performed to evaluate the resectability of HCCA, including tumor size, involvement of surrounding blood vessels, and extent of longitudinal involvement. A 640-CT scan was conducted to assess the future liver remnant (FLR), with preservation of at least 40%. FLR function was assessed using a liver function test and indocyanine green (ICG) assay. For patients with elevated serum total bilirubin (TB) levels (> 80 μmol/l), percutaneous trans-hepatic biliary drainage (PTBD) was performed before the operation to relieve biliary tract obstruction.

### Surgical procedure

**Figure 2:**
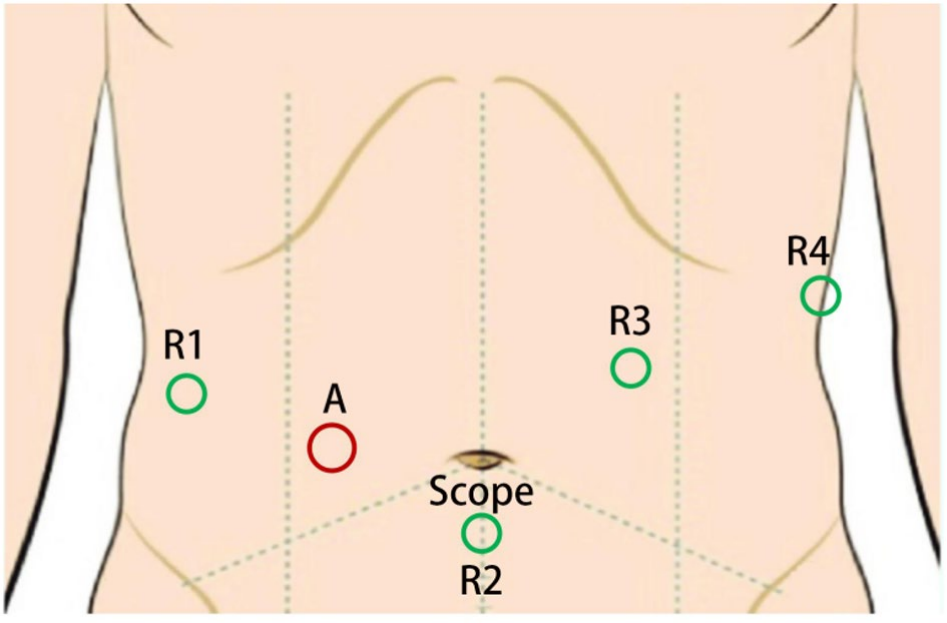
Trocar placement in robotic radical resection for hilar cholangiocarcinoma. R1, 8-mm trocar for the first robotic arm; R2, 8-mm trocar for the second robotic arm with scope; R3, 8-mm trocar for the third robotic arm; R4, 8-mm trocar for the fourth robotic arm; A, 12 mm trocar for assistant instruments.

The surgical procedure for patients admitted to the laparoscopic group was performed as previously described^6^. All robot-assisted procedures were performed using the da Vinci Xi Surgical System (Intuitive Surgical, Inc., Sunnyvale, USA). An example of a left hemihepatectomy was used to show anatomical schematic diagrams of RRRH. Patients undergoing robotic surgery were placed in the reverse Trendelenburg position, and a five-port method was used. The position of trocars is shown in Fig. 2. The surgical procedure was as follows.

**Figure 3:**
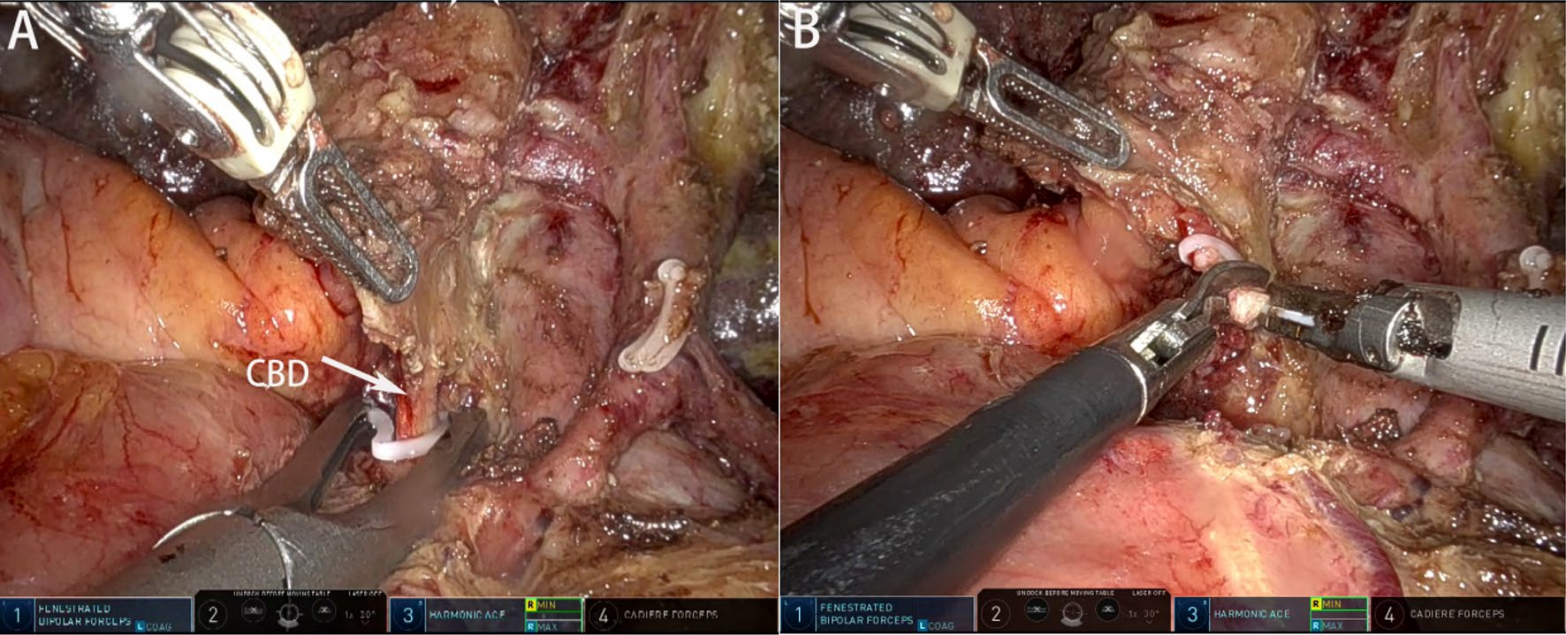
Exposure and management of common bile duct. (**A**) Ligation of distal common bile duct with Hem-o-lok clip. (**B**) Lower edge of the common bile duct was dissected and sent to the intraoperative pathological examination.
- The hepatoduodenal ligament was skeletonized to remove all soft tissues and lymph nodes starting from the common hepatic artery in a clockwise direction, including Groups 5, 7, 8, and 12 lymph nodes, following the previous description^16^. The common hepatic artery, proper hepatic artery, and portal vein were adequately isolated (Fig. 4).

- Intraoperative ultrasonography was routinely performed to eliminate potential intrahepatic metastases. Exposition and transection of the bile duct at the upper border of the pancreatic head were performed to determine whether the margins were negative (Fig. 3).

**Figure 4:**
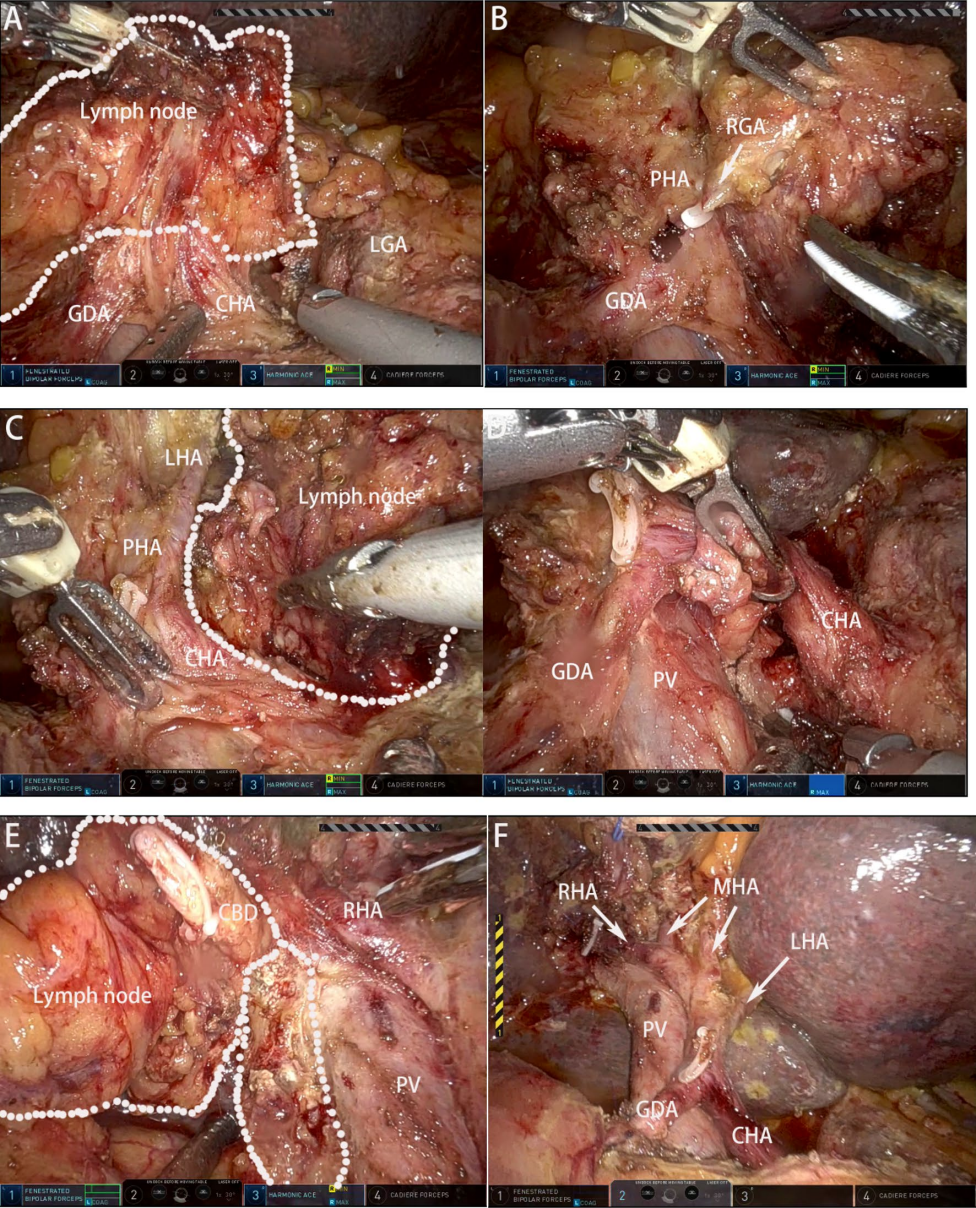
Regional lymph node dissection and skeletonization of the hepatoduodenal ligament. (**A**) Remove soft tissues and lymph nodes starting from the common hepatic artery. (**B**) Dissect the right gastric artery during cleaning 12 group lymph nodes. (**C**) Clean the left lymph node groups of the hepatoduodenal ligament to reveal the hepatic artery. (**D**) Cleaning lymph nodes around the celiac trunk. (**E**) Clean the right lymph node groups of the hepatoduodenal ligament combined with the lymph node of the 13 group. (**F**) After lymph node dissection. *GDA* gastroduodenal artery, *CHA* common hepatic artery, *LGA* left gastric artery, *RGA* right gastric artery, *PHA* proper hepatic artery, *LHA* left hepatic artery, *PV* portal vein, *RHA* right hepatic artery, *MHA* middle hepatic artery, *CBD* common bile duct.
- The left hepatic artery and left portal vein were occluded and divided successively, followed by cholecystectomy. The right hepatic duct was cut off at a soft point, and a negative margin was confirmed using frozen section pathology. Determination of the liver section relied on the middle hepatic vein orientation, which was determined by intraoperative ultrasound and ICG fluorescence imaging. Subsequently, a left hemihepatectomy combined with caudate lobe resection was performed en bloc (Fig. 5).

**Figure 5:**
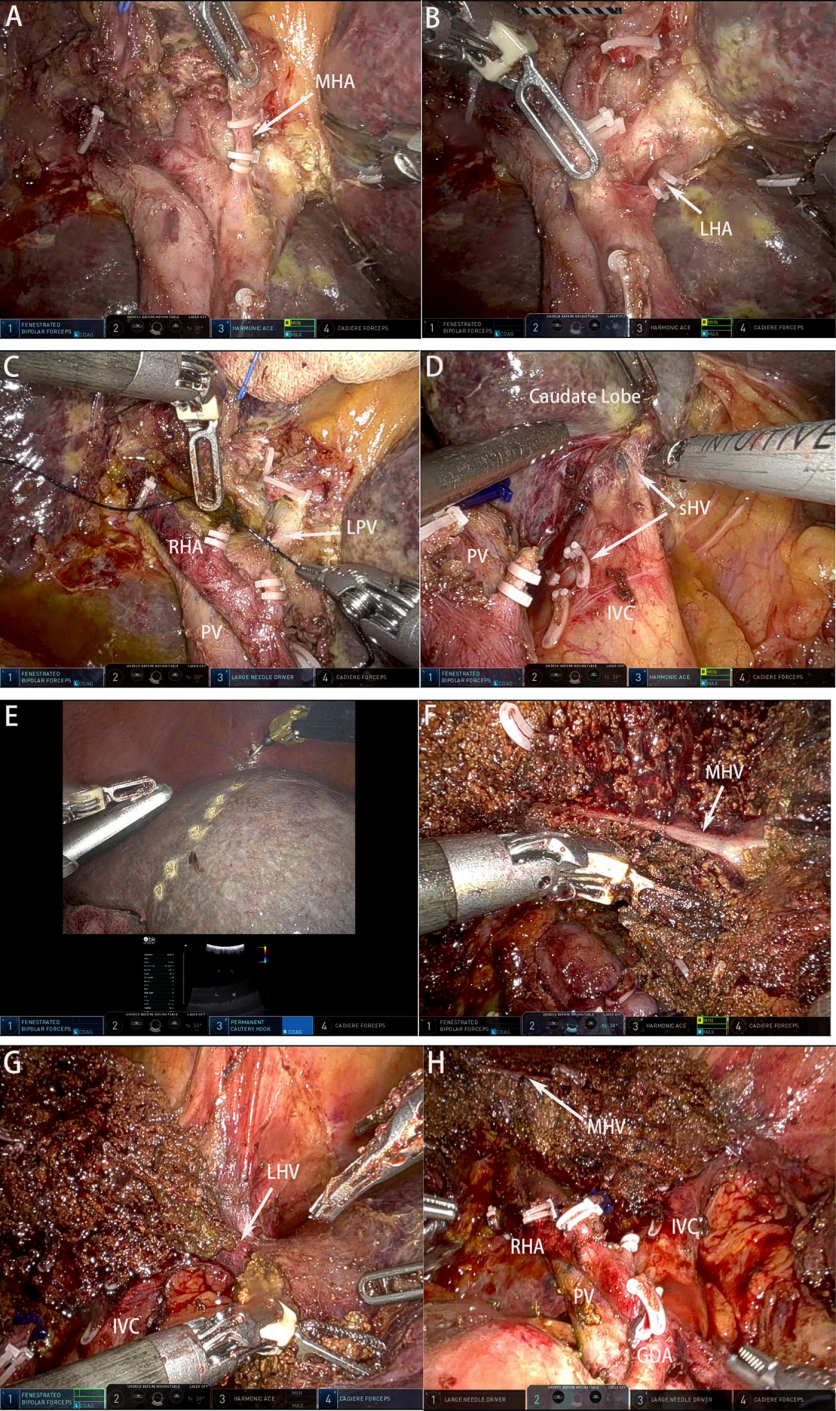
Procedure of left hemihepatectomy combined with caudate lobe resection. (**A**) Dissect the middle hepatic artery. (**B**) Dissect the left hepatic artery. (**C**) Ligate and dissect the left portal vein. (**D**) Separate the caudate lobe from the anterior of the inferior vena cava by processing the short hepatic vein. (**E**) Determine the liver section by relying on the ischemic boundary and intraoperative ultrasound positioning of the middle hepatic vein. (**F**) Dissect the liver parenchyma along the middle hepatic vein. (**G**) Expose the left hepatic vein. (**H**) After resection of left hemihepatectomy combined with caudate lobe. *MHA* middle hepatic artery, *LHA* left hepatic artery, *RHA* right hepatic artery, *LPV* left portal vein, *IVC* interior vena cava, *sHV* short hepatic vein, *PV* portal vein, *LHV* left hepatic vein, *MHV* middle hepatic vein, *GDA* gastroduodenal artery.
- Lastly, BER was performed using end-to-side anastomosis between the residual bile duct and Roux-en-Y jejunal loop without any stent, followed by intestinal anastomosis (Fig. 6). Ductoplasty of the intrahepatic bile ducts was performed before cholangiojejunostomy if necessary. Drainage tubes were routinely placed close to the hepaticojejunostomy site and cut the face of the liver.

**Figure 6:**
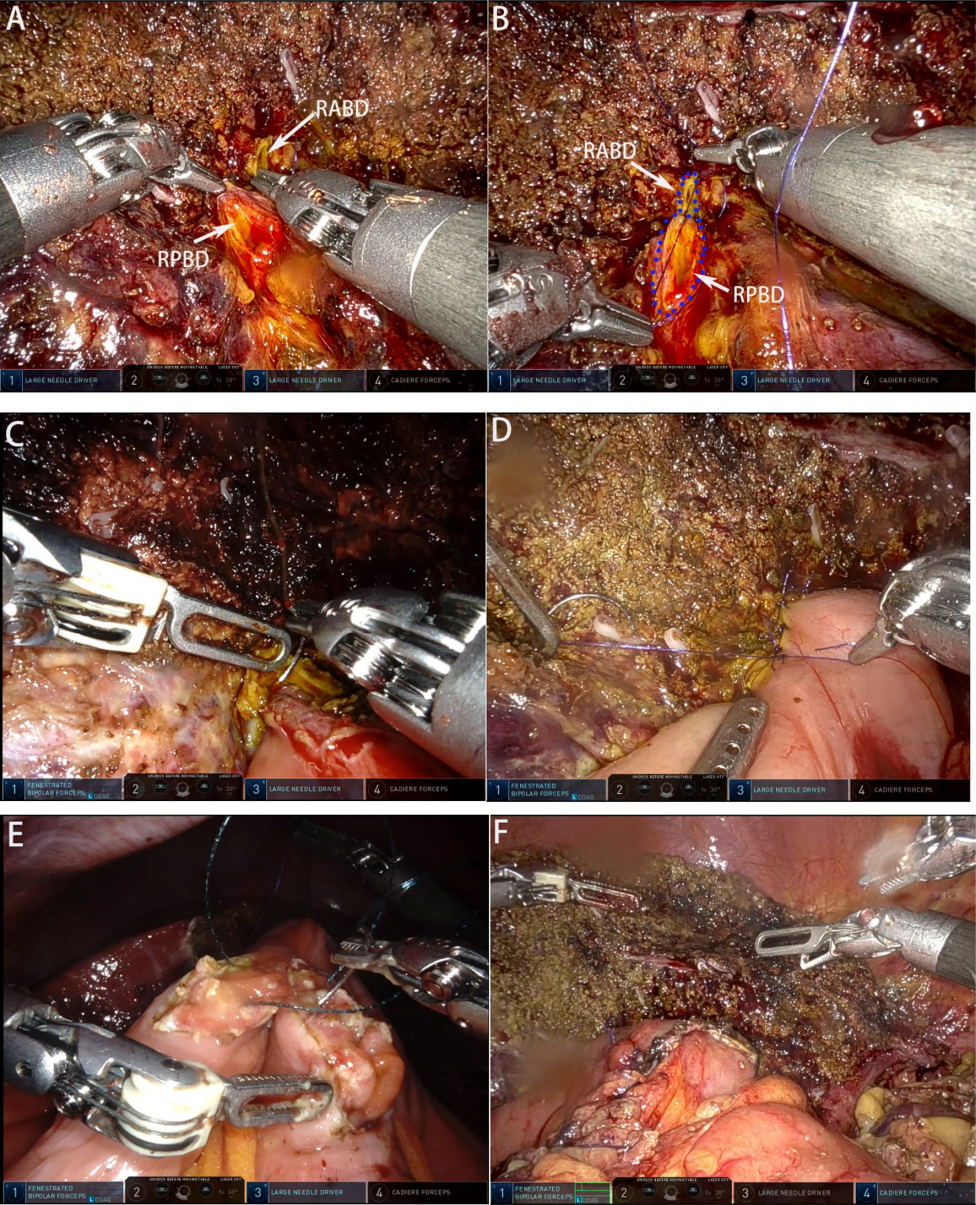
Procedure of left biliary-enteric reconstruction and intestinal anastomosis. (**A**) Expose the right anterior and posterior bile duct. (**B**) Perform the cholangioplasty of the right anterior and posterior bile duct with 5-0 PDS. (**C**) Complete the biliary-enteric reconstruction with a continuous suture of the posterior wall with a 4-0 barbed suture. (**D**) Complete the biliary-enteric reconstruction with interrupted suture of the anterior wall with 5-0 PDS. (**E**) Complete the intestinal anastomosis side to side with continuous sutures of the anterior and posterior wall with 3-0 barbed sutures. (**F**) After liver resection and biliary-enteric reconstruction. *RABD* right anterior bile duct, *RPBD* right posterior bile duct.

### Postoperative management

Laboratory blood examinations were performed based on the patient’s postoperative condition. Bilirubin levels in the drainage fluid, placed near the hepaticojejunostomy site, were measured, and bile leakage was defined according to the criteria proposed by the International Research Group on liver surgery^17^. Drainage tubes were removed if the enhanced CT scans showed no fluid accumulation in the peritoneal cavity or if there was no obvious fluid drainage after withdrawal. In cases where tubes cannot be removed because of continuous biliary leakage, they were retained for at least 2 months until a sinus tract was firmly formed and clamped for 1 week. Removal of the drainage tube was considered if the patient had no effusion, abdominal pain, or fever.

### PSM analysis

PSM, widely used in medical literature^18^, was performed to minimize deviations between the robotic and laparoscopic groups. Potential factors associated with the outcomes of radical resection of HCCA were matched, including sex, age, body mass index (BMI), American Society of Anesthesiologists (ASA) classification, TB, indocyanine green retention at 15 min (ICG R15), preoperative biliary drainage and reinfusion, tumor size, tumor stage, and Bismuth-Corlette classification. Each patient’s propensity score was calculated using a logistic regression, and patients were matched in a 1:2 ratio using the “nearest” approach to balance the baseline characteristics.

### Statistical analysis

SPSS statistical software (IBM SPSS, Inc., Chicago, IL, version 27) and GraphPad Prism (version 9.5.1) were used for statistical analysis. Categorical variables, presented as absolute numbers (percentage), were compared between groups using the χ^2^ test or Fisher’s exact test. Normally distributed continuous variables, presented as mean ± standard deviation (SD), were compared between groups using Student’s t-test. Continuous variables that did not follow a normal distribution are presented as medians and were analyzed using the Mann–Whitney U test. A P-value < 0.05 was considered statistically significant.

### Ethical approval

Written informed consent was obtained from all the enrolled patients. This study was approved by the Ethics Committee of Zhejiang Provincial People’s Hospital and followed the principles of the Declaration of Helsinki.

## Results

## Baseline and pathological characteristics before and after PSM

A total of 46 patients underwent radical resection for HCCA, 10 were treated with robot-assisted resection, and 36 underwent laparoscopic resection. None of the patients in either group required conversion to laparotomy. The clinicopathological features of the patients are summarized (Table 1). No significant differences were observed between the RRRH and LRRH groups regarding sex, age, BMI, ASA classification, preoperative biliary drainage, bile reinfusion, ICG R15, Bismuth–Corlette classification, tumor size, or T stage. The average TB level in the RRRH group was lower than that in the LRRH group (P = 0.047). All patients underwent hemihepatectomy combined with caudate lobectomy, regional lymphadenectomy, extrahepatic bile duct resection, and EBR, except for those with Bismuth type I, who underwent extrahepatic bile duct resection only. A total of 28 patients in both groups did not undergo bile reinfusion because of patient rejection.

**Table 1:** Baseline and pathological characteristics before and after PSM between two group.

	Before PSM			After PSM		
	RRRH group (n = 10)	LRRH group (n = 36)	P-value	RRRH group (n = 10)	LRRH group (n = 20)	P-value
Gender, n (%)			0.707			0.690
Male	6 (60.0%)	25 (69.4%)		6 (60.0%)	14 (70.0%)	
Female	4 (40.0%)	11 (30.6%)		4 (40.0%)	6 (30.0%)	
Age, year, mean (SD)	64.0 ± 8.8	66.1 ± 9.6	0.536	64.0 ± 8.8	65.6 ± 8.8	0.643
BMI, kg/m^2^, median	23.5 (19–30)	24 (18–30)	0.646	23.5 (19–30)	24 (18–30)	0.681
ASA classification ≥ III, n (%)	2 (20.0%)	5 (13.9%)	0.636	2 (20.0%)	3 (15.0%)	> 0.999
Total bilirubin, µmol/mL, mean (SD)	168.2 ± 87.7	239.4 ± 100.0	0.047	168.2 ± 87.7	218.3 ± 94.0	0.221
Preoperative PTBD, n (%)	6 (60.0%)	29 (80.6%)	0.220	6 (60.0%)	16 (80.0%)	0.384
Bile reinfusion, n (%)	4 (40.0%)	14 (38.9%)	1.000	4 (40.0%)	7 (35.0%)	> 0.999
ICG R15, %, median	8.8 (3.3–14.1)	8.0 (1.2–18.4)	0.783	8.8 (3.3–14.1)	8.4 (1.2–13.6)	0.713
Bismuth type, n (%)			> 0.999			> 0.999
I	0 (0.0%)	1 (2.78%)		0 (0.0%)	0 (0.0%)	
II	1 (10.0%)	5 (13.9%)		1 (10.0%)	1 (5.0%)	
III	7 (70.0%)	20 (53.6%)		7 (70.0%)	14 (70.0%)	
IV	2 (20.0%)	10 (27.8%)		2 (20.0%)	5 (25.0%)	
Tumor size, cm, mean (SD)	2.1 ± 0.7	2.0 ± 0.9	0.821	2.1 ± 0.7	1.8 ± 0.6	0.322
AJCC T stage, n (%)			0.636			> 0.999
T1 + T2	8 (80.0%)	31 (86.1%)		8 (80.0%)	17 (85.0%)	
T3 + T4	2 (20.0%)	5 (13.9%)		2 (20.0%)	3 (15.0%)	

*PSM* propensity score matching, *BMI* body mass index, *ASA* American Society of Anesthesiologists, *PTBD* percutaneous trans-hepatic biliary drainage, *ICG R15* indocyanine green retention at 15 min, *AJCC* American Joint Committee on Cancer.

### Intraoperative parameters and postoperative outcomes

Table 2 shows the intraoperative parameters and postoperative outcomes in the two groups after PSM. The surgical strategy for HCCA in this study was based on the Bismuth–Corlette classification. Patients with bismuth types II and IIIb underwent left hemihepatectomy (LH) with caudate lobectomy (CL). Bismuth type IIIa was treated with a right hemihepatectomy (RH) and CL. Bismuth type IV underwent typical or extended LH or RH depending on the extent of tumor invasion into the left and right hepatic ducts, the residual liver volume, and ICG R15. Lobus quadratus resection (segments IVb + V) combined with CL was considered appropriate for patients with an FLR of less than 40%. No significant difference in the surgical type was observed between the two groups. Interestingly, although there was no significant difference in the total operation time between the two groups (P = 0.931), the RRRH group’s liver resection time was significantly longer than that of the LRRH group (139.5 ± 38.8 vs 108.1 ± 35.8 min, P = 0.036), possibly owing to the proficiency of the surgeon and frequent replacement of hemostatic devices in the RRRH group. The RRRH group showed less intraoperative blood loss [200 (50–500) vs 310 (100–850) ml]), potentially caused by a more delicate operation; however, there was no significant difference (p = 0.109). Six patients in both groups did not undergo a negative bile duct margin because of extensive tumor invasion. Blood reinfusion, TNLE, resection margin, and the incidence of severe morbidity (CD ≥ III) were comparable between the groups. No significant difference was observed between the two groups in postoperative 30-day and 90-day mortality or postoperative hospital stay.

**Table 2:** Intraoperative parameters and postoperative outcomes in two groups.

	RRRH group (n = 10)	LRRH group (n = 20)	P-value
Surgical type, n (%)			0.817
LH + CL	5 (50.0%)	12 (60.0%)	
RH + CL	3 (30.0%)	4 (20.0%)	
LQR + CL	2 (20.0%)	4 (20.0%)	
Operation time, min, mean (SD)	384.6 ± 72.2	373.5 ± 67.9	0.931
Liver resection time, min, mean (SD)	139.5 ± 38.8	108.1 ± 35.8	0.036
Intraoperative blood loss, ml, median	200 (50–500)	310 (100–850)	0.109
Blood transfusion, n (%)	1 (10.0%)	6 (30.0%)	0.372
TNLE, median	11 (6–31)	10.5 (6–29)	0.559
Resection margin, n (%)			0.829
R0	8	16	
R1	1	3	
R2	1	1	
Postoperative hospital stay, days, median	9.3 ± 2.2	11.1 ± 3.8	0.166
Serve morbidity (CD ≥ III), n (%)	2	6	0.682
30-day mortality, n (%)	0	0	> 0.999
90-day mortality, n (%)	0	0	> 0.999

*LH* left hemihepatectomy, *RH* right hemihepatectomy, *CL* caudate lobectomy, *LQR* lobus quadratus resection, *TNLE* total number of lymph nodes examined, *CD* Clavien–Dindo classification.

### Biliary-enteric reconstruction (BER) and relative complication

Details of the outcomes of BER and related complications are summarized in Table 3. According to different surgical types, the number of residuals ranged from 1 to 5. The average number of the residuals in the RRRH and LRRH groups was 2.6 ± 1.3 and 2.7 ± 1.2, respectively (P = 0.795). The average residual diameter was comparable between the groups (P = 0.138). To minimize the number of hepaticojejunostomies, cholangioplasty is usually performed on the adjacent bile duct stumps. Choledochojejunostomies ranged from 1 to 3, with the same mean of 1.6 ± 0.7 in both groups (P = 0.965).

**Table 3:** Biliary-enteric reconstruction and relative complications in two groups.

	RRRH group (n = 10)	LRRH group (n = 20)	P-value
Number of residuals, mean (SD)	2.6 ± 1.3	2.7 ± 1.2	0.795
Diameter of residuals, cm, mean (SD)	5.0 ± 2.0	4.8 ± 1.6	0.138
Number of anastomotic, mean (SD)	1.6 ± 0.7	1.6 ± 0.7	0.965
Anastomosis manner, n (%)			> 0.999
IS	6 (42.9%)	8 (29.6%)	
CS	5 (35.7%)	14 (51.9%)	
CSPW + ISAW	3 (21.4%)	5 (18.5%)	
Anastomosis time, min, mean (SD)	38.4 ± 13.6	59.1 ± 25.5	0.024
Time ratio of EBR, %,, mean (SD)	9.9 ± 2.8	15.4 ± 4.8	0.001
Tube drawing, days, mean (SD)	6.7 ± 4.4	12.1 ± 11.7	0.019
Complications related to BER, n (%)			
Bile leakage	1 (10.0%)	8 (40.0%)	0.204
A	1 (10.0%)	3 (15.0%)	> 0.999
B	0 (0.0%)	5 (25.0%)	0.140
Peritonitis	0 (0.0%)	1 (5.0%)	> 0.999
Anastomotic stenosis	1 (10.0%)	6 (30.0%)	0.372
Anastomotic calculus	0 (0.0%)	3 (15.0%)	0.532
Bleeding	0 (0.0%)	0 (0.0%)	> 0.999
Death	0 (0.0%)	0 (0.0%)	> 0.999

*IS* intermittent suture, *CS* continuous suture, *CSPW* continuous suture on the posterior wall, *ISAW* intermittent suture on the anterior wall, *BER* biliary-enteric reconstruction.

The hepaticojejunostomy suture method was used based on the diameter of the residual bile duct. Small bile ducts with a diameter of less than 1 mm were ligated directly. For intrahepatic bile ducts with diameters less than 5 mm (or after cholangioplasty), end-to-side anastomoses were performed through intermittent sutures of the anterior and posterior walls with 5-0 PDS. For a diameter of 5–8 mm, the posterior wall was continuously sutured with 4-0 barbed suture, and the anterior wall was sutured intermittently with 5-0 PDS, respectively. Both the anterior and posterior walls were sutured continuously using 4-0 barbed suture in patients with diameters of > 8 mm. No statistically significant difference was observed between the two groups in terms of anastomosis (P > 0.999). The average time of BER in the RRRH and LRRH groups was 38.4 ± 13.6 and 59.1 ± 25.5 min, respectively (P = 0.024), accounting for 9.9 ± 2.8% and 15.4 ± 4.8% of the total operation time in each group (P = 0.001). This indicates a significantly lower time consumption ratio for completing the BER in the RRRH group.

One of the most common complications after EBR is postoperative bile leakage, with nine cases observed in the two groups: one from the RRRH group grade as A, and eight cases from the LRRH group, including three grade A and five grade B; no grade C was observed. Only one patient with grade B developed local peritonitis owing to obstructed drainage, cured by CT-guided percutaneous catheter drainage. All other patients were cured with continuous abdominal drainage without additional treatment. No difference was observed in the incidence of bile leakage in the two groups (p = 0.204), and the mean days of tube drawing in the RRRH groups was shorter than that in the LRRH group (6.7 ± 4.4 vs 12.1 ± 11.7 days, P = 0.019). One case of cholangiointestinal anastomotic stricture occurred in the RRRH group and six in the LRRH group, presenting with recurrent fever confirmed by MRCP from 3 months to 2 years after surgery. Among the six cases in the LRRH group, three combined with stone formation were resolved by percutaneous transhepatic choledochoscopy.

## Discussion

Radical resection of HCCA is widely regarded as the most challenging procedure in biliary surgery, requiring extensive liver resection, including caudate lobe resection, whole regional lymph node dissection, high bile-enteric anastomosis, and vascular resection and reconstruction. With the continuous advancements in surgical theory, technology, and the rapid development of surgical instruments, LRRH has been performed in some large hepatobiliary surgery centers; Xiong^19^ compared Laparoscopic and open groups, revealing similar intraoperative bleeding volume, number of lymph nodes retrieved, bile leakage, and liver failure between the two groups. Ma^20^ found no significant differences in surgical outcomes between open and laparoscopic surgery groups; however, the overall survival rate and disease-free survival rates were significantly higher in the open surgery group. Zhang^6^ reported that laparoscopic radical resection of HCCA was significantly better than traditional laparotomy in terms of intraoperative blood loss and postoperative hospitalization time, with no statistically significant difference in postoperative long-term survival. However, inherent limitations of laparoscopy, such as inflexible operation, poor device mobility, fulcrum effect, anti-ergonomics, and a long learning curve^21,22^, limit its wide application in HCCA.

In contrast, robotic surgery systems can overcome the disadvantages of traditional laparoscopy in complex anatomical and anastomotic surgeries and may have potential advantages in minimally invasive surgery for HCCA. Although robotic-assisted radical resection for HCCA was reported as early as 2010^23^ and has been performed in many hepatobiliary surgery centers^24–27^, the application of robots in HCCA remains controversial. Xu^25^ reported ten cases of robotic surgery for HCCA, with longer operative times and greater blood loss, and concluded that robotic surgery may not be suitable for the treatment of HCCA. Huang^26^ evaluated the short-term outcomes of robotic resection for HCCA and found that intraoperative bleeding, transfusion rate, and postoperative morbidity were often lower in the robotic group than in the open group, although these differences were not statistically significant. Cou^27^ compared robotic and open surgery and revealed that there was no difference in short- or long-term effects after HCCA resection between the two groups. The consistent results were obtained in this study; the RRRH group showed less intraoperative blood loss [ (200 (50–500) vs 310 (100–850) ml, P = 0.109], postoperative hospital stay (9.3 ± 2.2 vs 11.1 ± 3.8 days, P = 0.166), similar total operation time and complications compared with the LRRH group. Accordingly, in selected patients, the robotic surgical method for HCCA is technically feasible, and enthusiasm should be encouraged.

The most important advantage of the robotic surgical system in the radical resection of HCCA is mainly reflected in the high biliary tract reconstruction. Complex Roux-en-Y hepaticojejunostomy is considered a difficult and important rate-limiting step in LRRH. First, the obstruction caused by the hilar liver tumor generally thins the tube wall of the distal dilated bile duct, and the tissue of the bile duct wall is often fragile owing to impaired liver function, nutritional status, and inflammatory response. Limited suture angle caused by the straight instruments of laparoscopy often leads to anastomotic tear in the reconstructive process because of uncontrollable suture strength. Second, although the negative bile duct margin could finally be confirmed by rapid intraoperative pathological examination, it was still difficult to determine the high bile duct disconnection point during surgery. Whether the texture of the bile duct was soft could be judged approximately by the tactile feedback of the hand in open surgery, which was absent in laparoscopic surgery and always led the surgeon to unconsciously choose higher bile ducts for negative margins, resulting in thinner bile ducts at the anastomotic site objectively.

Additionally, most patients undergoing radical resection for HCCA had two or more anastomoses. The previously completed anastomosis occupied space for the subsequent anastomosis, often challenging and increasing the time and inaccuracy of the anastomosis. Therefore, overcoming the inherent angle defects of laparoscopy to achieve satisfactory anastomosis remains difficult, even for surgeons with extensive experience and proficient suture techniques. This difficulty may result in postoperative biliary fistula, anastomotic stenosis, and other serious complications. Liu^28^ compared BER between laparoscopic and open radical resection for HCCA and found a significant difference in the anastomosis time (65.67 ± 21.53 vs 42.5 ± 19.77 min, P < 0.05) and BER time ratio (15.08 ± 3.64% vs 11.76 ± 2.54%, P < 0 0.05), with longer times observed in the laparoscopic group mainly owing to the complexity of laparoscopic BER procedures. However, in a systematic review, Guerra^29^ suggested that robotic surgery has become a treatment option for biliary tract surgery in large clinical centers. The benefits of the robotic technology have accelerated this shift. Surgeries requiring microanastomosis and high precision, such as Roux-en-Y hepaticojejunostomies, are the best indications for the robotic approach. In addition, the robotic surgical system ensures that the surgeon is in a comfortable sitting position for suturing, which subjectively improves operative accuracy, facilitating safe and reliable biliary reconstruction. Our research confirmed this conclusion; in our study, the BER completion time and its ratio to total operative time were significantly shorter in the RRRH group than in the LRRH group.

Limited research exists on robot-assisted Roux-en-Y hepaticojejunostomy.^28,30,31^. In this study, a mesentery with fewer blood vessels was selected for appropriate cutting to ensure that the raised intestinal loop had sufficient length. Anastomosis was performed through the anterior mesentery of the transverse colon, and the transverse mesocolon posterior approach was used only when tension was applied. Regarding the method of biliary intestinal suture in the study, intermittent suture, continuous suture, or a combination of the two methods were chosen according to the diameter of the bile duct and the thickness of the bile duct wall. For bile ducts with a diameter less than 5 mm, intermittent suture on both the anterior and posterior walls was appropriate to prevent tearing and postoperative stenosis effectively. When the diameter was greater than 8 mm, continuous sutures had an obvious advantage in suturing speed and economical surgical operation time. The combination of intermittent and continuous sutures was suitable for bile ducts with a diameter from 5 to 8 mm. Continuous sutures can be used for thick bile duct walls, whereas intermittent sutures are suitable for thick bile duct walls. This study showed that the incidence of biliary fistula (10% vs. 40%, P = 0.204), anastomotic stenosis (10% vs. 30%, P = 0.372), and stone formation (0% vs. 10%, P = 0.532) were lower in the RRRH group, indicating that robotic surgery was better in terms of the quality of biliary-enteric anastomosis and had a lower incidence of long-term complications, attributed to more refined robotic suturing.

Although the application prospects of robotic surgery in HCCA have been acknowledged, data on the long-term outcomes of RRRH are lacking. A previous study reported a median disease-free survival of 15.5 months in patients with HCCA who underwent robot-assisted radical resection^25^. However, other studies on robotic radical resection for HCCA have only analyzed the short-term results^14,32^. Therefore, additional randomized controlled trials are required to evaluate long-term efficacy.

To our knowledge, this is the only study to compare BER-related results between robotic and laparoscopic surgery for HCCA. However, this study had some limitations that must be noted. First, it was a single-center, retrospective clinical study with a relatively small sample size, which may have led to biased results. Second, owing to the relatively late initiation of RRRH at our center, data on long-term efficacy are insufficient. The effectiveness of RRRH requires further exploration in large-scale prospective multicenter trials.

## Data Availability

The datasets used and analyzed in this study are available from the corresponding author upon reasonable request.
